# Magnetic, biocompatible FeCO_3_ nanoparticles for T2-weighted magnetic resonance imaging of in vivo lung tumors

**DOI:** 10.1186/s12951-022-01355-3

**Published:** 2022-03-25

**Authors:** Suresh Thangudu, Chun-Chieh Yu, Chin-Lai Lee, Min-Chiao Liao, Chia-Hao Su

**Affiliations:** 1 Institute for Translational Research in Biomedicine, Kaohsiung Chang Gung Memorial Hospital, Kaohsiung, 833 Taiwan; 2 Department of Biomedical Imaging and Radiological Sciences, National Yang Ming Chiao Tung University, Taipei, 112 Taiwan; 3 Center for General Education, Chang Gung University, Taoyuan, 333 Taiwan

**Keywords:** Metal carbonates, Magnetic resonance, T2 weighted contrast, In vivo lung tumors, Biosafety

## Abstract

**Background:**

Late diagnosis of lung cancer is one of the leading causes of higher mortality in lung cancer patients worldwide. Significant research attention has focused on the use of magnetic resonance imaging (MRI) based nano contrast agents to efficiently locate cancer tumors for surgical removal or disease diagnostics. Although contrast agents offer significant advantages, further clinical applications require improvements in biocompatibility, biosafety and efficacy.

**Results:**

To address these challenges, we fabricated ultra-fine Iron Carbonate Nanoparticles (FeCO_3_ NPs) for the first time via modified literature method. Synthesized NPs exhibit ultra-fine size (~ 17 nm), good dispersibility and excellent stability in both aqueous and biological media. We evaluated the MR contrast abilities of FeCO_3_ NPs and observed remarkable T2 weighted MRI contrast in a concentration dependent manner, with a transverse relaxivity (r2) value of 730.9 ± 4.8 mM^−1^ S^−1^at 9.4 T. Moreover, the r2 values of present FeCO_3_ NPs are respectively 1.95 and 2.3 times higher than the clinically approved contrast agents Resovist^®^ and Friedx at same 9.4 T MR scanner. FeCO_3_ NPs demonstrate an enhanced T2 weighted contrast for in vivo lung tumors within 5 h of post intravenous administration with no apparent systemic toxicity or induction of inflammation observed in in vivo mice models.

**Conclusion:**

The excellent biocompatibility and T2 weighted contrast abilities of FeCO_3_ NPs suggest potential for future clinical use in early diagnosis of lung tumors.

**Graphical Abstract:**

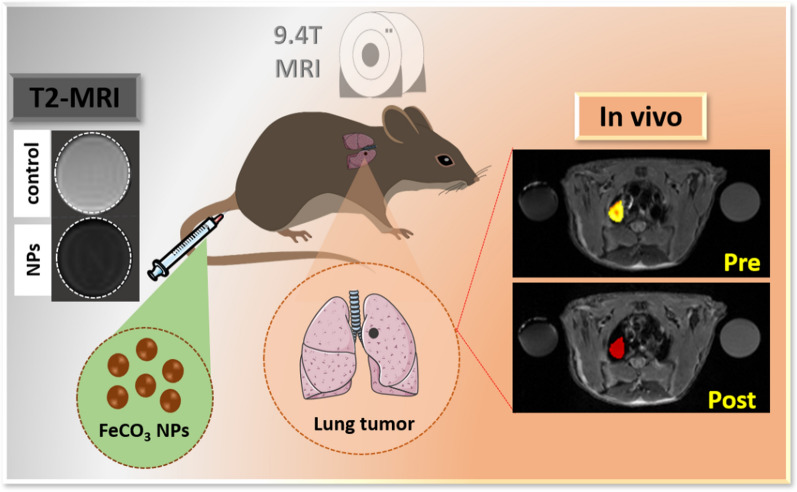

**Supplementary Information:**

The online version contains supplementary material available at 10.1186/s12951-022-01355-3.

## Background

Lung carcinoma causes high rates of mortality worldwide, with nearly 2.1 million diagnoses and 1.8 million deaths in 2018 [1]. Despite the availability of surgical removal, cytotoxic chemotherapy, and radiation therapy, long-term survival rates are still below 15% [2]. To date, surgery remains the primary intervention for early diagnosed lung cancers [3]. Unfortunately, surgery is less effective in late-stage lung cancer patients due to the uncontrolled invasion and metastasis of lung tumors and the difficulty of complete excision without leaving microscopic tumors behind. More than 20% of early-diagnosed lung cancers are inoperable due to advanced patient age, severely impaired lung function, and other comorbidities. The key factor in higher mortality in lung cancer patients is late diagnosis. This raises an urgent need for an alternative approach to successfully diagnosis lung tumors in an early stage. To this end, imaging modalities such as X-ray computed tomography (CT), positron emission tomography (PET), and magnetic resonance imaging (MRI) offer significant hope for efficient clinical tumor detection [4]. Among these imaging modalities, magnetic resonance imaging (MRI) has a critically important role in molecular imaging and clinical diagnosis because it is non-invasive, offers excellent soft tissue contrast and can produce images with high spatial and temporal resolution (Additional file 1: Table S1) [5, 6]. However, approximately 35% of clinical MR scans require contrast agents to further improve sensitivity and diagnostic accuracy [7, 8]. Paramagnetic gadolinium (Gd^3+^) chelates have been extensively used as T1 weighted contrast agents in MRI to further enhance diagnostic accuracy with significant clinical outcomes [9]. However, the United States Food and Drug Administration (USFDA) has warned against the usage of Gd-based contrast agents because of the potential risk of side effects such as nephrogenic systemic fibrosis and kidney dysfunction [10, 11]. Therefore, it is highly desirable to develop a novel alternative to Gd^3+^ chelates.

**Scheme 1:**
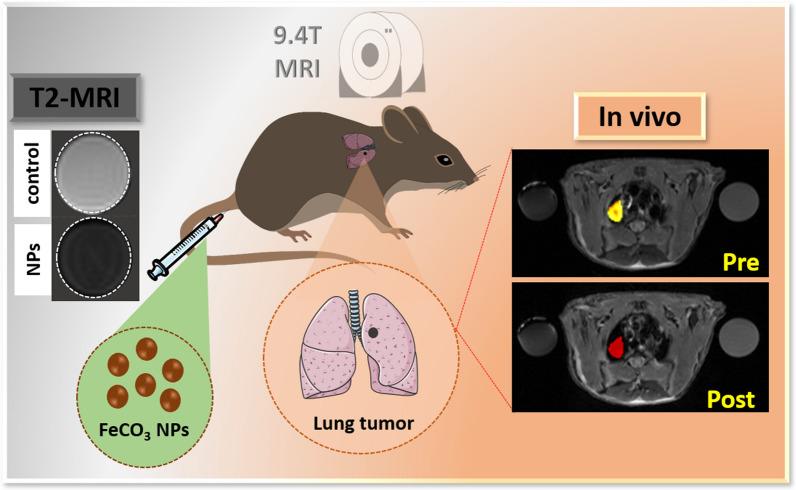
Schematic illustration of in vivo MR imaging of lung tumors on FeCO_3_ NPs

Increased research attention has focused on superparamagnetic iron oxide nanoparticle (SPION)-based T2 contrast agents due to their nontoxicity and biodegradability [12, 13]. The USFDA approved the Resovist^®^ and Friedx formulations for use as T2 weighted MR contrast agents in clinical applications [14, 15], but they were subsequently recalled following a re-evaluation of safety and efficacy [16]. To address the safety issues, surface modification ligands such as polyethylene glycol (PEG), polysaccharides, peptides or dextran were coated on SPIONs and examined at both in vitro and in vivo levels [17–19]. Despite offering good biocompatibility, such a coating on SPION may increase the hydrodynamic size and diminish the efficacy of the particles’ magnetic contrast abilities [20]. Thus, it is imperative to develop a novel T2 weighted contrast agent with high relaxivity, sensitivity and excellent biocompatibility for high-performance MRI diagnosis. Recently, metal carbonates, specifically calcium carbonate (CaCO_3_)-based nanomaterials (NMs) have been widely used in biomedical applications, offering improved biocompatibility, biodegradability, and biosafety [21–23]. CaCO_3_ coated/co-doped magnetic nanoparticles were fabricated and used in an MRI-guided theranostic platform for treating tumors with significant outcomes [24–26]. However, surface coated/co-doped CaCO_3_ will decompose under the acidic pH conditions in tumors, raising serious questions about the intrinsic toxicity and biocompatibility of loaded NMs. Thus, a direct magnetic metal carbonate nanoformulation offers a promising alternative to overcome the existing limitations and minimize side effects. As a result, manganese metal carbonates (MnCO_3_) such as MnCO_3_@PDA [27], MnCO_3_@PEA [28] were successfully utilized as T1 weighted MR contrast agents for in vivo subcutaneous tumors, offering acidic pH-triggered contrast ability with no cytotoxicity. However, surface coating ligands on the nanoformulations are still a considerable factor for MR contrast properties. In contrast, direct magnetic metal carbonates as a T2-weighted MR contrast agents have not yet been reported in the literature. The present work reports the first fabrication of ultra-fine FeCO_3_ NPs for T2 weighted in vivo real-time MR imaging of lung tumors. The synthesized FeCO_3_ NPs exhibit enhanced T2 weighted contrast abilities with an r2 value of 730.9 ± 4.8 mM^−1^ S^−1^, a significant improvement on clinically approved contrast agents. Furthermore, the proposed FeCO_3_ NPs offer remarkable T2-weighted enhancement in both in vitro and in vivo systems. Notably, tumor specific contrast of ≥ 85% was achieved within 5 h post injection of FeCO_3_ NPs via intravenous (iv) administration in lung tumors implanted in mice models. Furthermore, biosafety data reveals that proposed FeCO_3_ NPs are biocompatible with no significant toxic effects to the function of major organs such as liver or kidney. Overall, results suggest that the present biocompatible, non-toxic FeCO_3_ NPs are an excellent choice for future clinical detection of lung tumors. A schematic illustration of the in vivo T2-weighted MR imaging of lung tumors on as-synthesized FeCO_3_ NPs is shown in Scheme 1.

## Materials and methods

### Materials

Ferric chloride hexahydrate (FeCl_3_·6H_2_O), sodium carbonate (Na_2_CO_3_), and ascorbic acid (AA) were purchased from Sigma-Aldrich at highest available purity and used without further purification.

### Synthesis of FeCO_3_ NPs

Briefly, 4 mmol of FeCl_3_·6H_2_O was added to 50 mL distilled water and stirred for 30 min at room temperature. After dissolving completely, 24 mL of 0.5 M Na_2_CO_3_ aqueous solution was added slowly. After that, 5.3 mmol of AA was added to the above solution and stirred for 30 min at room temperature. After 30 min, the reaction solution was transferred into a 100 mL Teflon lined autoclave and heated for 12 h at 160 ℃, after which the reaction was cooled to room temperature and washed with ethanol and water several times, followed by drying at 60 °C for 6 h, producing micrometer-scale FeCO_3_. To achieve a nano-sized FeCO_3_, the resulting particles were dispersed in deionized water and centrifuged at 6000 rpm for 5 min. Finally, a supernatant solution containing FeCO_3_ NPs were collected and further stored at room temperature for further applications.

### Evaluation of T1/T2 contrast abilities of FeCO_3_ NPs

Aqueous solutions with different concentrations of FeCO_3_ NPs (between 0 to 0.1 mM, concentration respective to Fe) were diluted with a 1:1 water agar mixture and subsequently evaluated for T1 and T2-weighted MR imaging using a 9.4 T MRI instrument with a head coil (Biospec 94/20, Bruker, Ettingen, Germany). Furthermore, r1 and r2 relaxivity values were obtained by plotting the relaxation time 1/T1/T2 (s^−1^) vs the molar concentration of Iron (mM).

### In vitro cytotoxicity assay of FeCO_3_ NPs in normal cells

MRC-5 normal cells (human fetal lung fibroblast cells) were seeded in 96-well plates at a density of 1 × 10^4^ cells per well. After 24 h, different concentrations (0, 10, 25, 50, 75, 100, 150, 200 μg/mL) of FeCO_3_ NPs were added to the cells and incubated for 24 and 48 h. Thereafter, 20 μL of CCK-8 reagent was added into each well and further incubated for 4 h at 37 °C and 5% CO_2_. Absorbance (at 450 nm) of each well was then recorded using an ELISA reader (Thermo scientific, Hudson, NH). Mean and standard deviation (SD) were calculated from three parallel readings.

### Hemolysis assay

To collect the red blood cells (RBC), 5 mL of mice blood was obtained and centrifuged at 2500 rpm (5 min) and then washed with PBS three times. The resulting RBCs were suspended in 40 mL of PBS and stored at 4 °C for further usage. Later, different concentrations of the FeCO_3_ NPs were added to 0.5 mL of the RBC solution and incubated for 2 h at room temperature. For comparison, PBS and deionized water were respectively used as negative and positive controls. After 2 h of incubation, all samples were centrifuged, and the absorption of the resulting supernatants was measured at 561 nm. From the absorption results, we estimated the hemolysis percentage using following equation: Hemolysis rate = [(OD_samples_ – OD_negative_)/(OD_positive_ – OD_negative_) × 100%] [29].

### In vitro MR contrast abilities of FeCO_3_ NPs

Different concentrations of FeCO_3_ NPs (0, 15, 70, 140 mM, respective to Fe ion) were incubated with the pre-seeded lung cancer cells (CL1-5-luc/GFP) (3 × 10^5^ cells per well). After 24 h of incubation, the cell pellets were washed with PBS and dispersed in the 1:1 agarose deionized water mixture solution to evaluate the T2-weighted MR imaging using a 9.4 T MRI instrument with a head coil (Biospec 94/20, Bruker, Ettingen, Germany).

### Animal models

NOD/SCID nude mice (male, 8–10 weeks old) and BALB/c mice (male, 8–10 weeks old) were purchased from the Experimental Animal Center of the National Science Council, Taiwan. The mice were housed under temperature control (24–25 °C) and a 12-h light–dark cycle (lights on at 07:00) at the Chang Gung Memorial Hospital Laboratory Animal Center which is accredited by the Association for Assessment and Accreditation of Laboratory Animal Care.

### Establishment of lung tumors in animal models

In vivo imaging studies were performed on NOD/SCID nude mice (male, 8–10 weeks old). All experimental protocols involving live animals were reviewed and approved by the Institutional Animal Care and Use Committee (IACUC No. 2021031802) of Chang Gung Memorial Hospital and were performed in accordance with the Animal Protection Regulations of the Council of Agriculture, Executive Yuan (R.O.C.) and the guidelines of National Research Council (USA) for the care and use of laboratory animals. For lung tumor implantation, mice were anesthetized using an intraperitoneal injection of Zoletil^®^ (Virbac, France) and placed in a face-up position. A solution of 10 µL RPMI-1640 medium (5 × 10^4^ CL1-5-Luc/GFP cells) and 10 µL Basement Membrane Matrix (BD, USA) was directly injected (3 mm depth) into the right lung of the mice via BD Insulin Syringes 30G 3/10 cc (BD, USA).

### IVIS imaging system for tumor monitoring

Fourteen days following tumor implantation, bioluminescence flux was monitored to assess tumor growth by intraperitoneal injection of D-Luciferin (Caliper Life Sciences). Ten minutes following d-Luciferin injection, mice were subjected to IVIS bioluminescence (emission = 560 nm) imaging (PerkinElmer, Waltham, MA, USA) and the resulting images were analyzed using living imaging software with a pseudo color image representing the tumor.

### Evaluating the T2 weighted MR efficacy of FeCO_3_ NPs in lung tumor animal models

Lung tumor-bearing mice were first anesthetized and an aqueous solution of FeCO_3_ NPs (10 mg Fe/kg) was intravenously injected into each mouse, followed by scanning with a 9.4 T MRI scanner (Biospec 94/20, Bruker, Ettingen, Germany) with a transmit-receive volume coil (75/40 mm). MR images were collected both pre- and post-injection (1 h, 3 h and 5 h). To obtain higher resolution T2-weighted images (both coronal and axial), we used the multislice turbo rapid acquisition with refocusing echoes (Turbo-RARE) sequence with the following parameters: field of view (FOV) = 35.0 × 35.0 mm; matrix size = 192 × 192; spatial resolution = 182 × 182 μm; slice thickness = 1 mm; effective echo time (TE) = 15 ms; echo time = 15 ms; repetition time (TR) = 1000 ms; rare facto = 2; refocusing flip angle = 180º; number of averages = 1; number of repetitions (NR) = 1. All images were acquired with respiration and ECG trigger using Amira software, version 2020.2.

### Histological examination

Five days following the administration of FeCO_3_, the animals were sacrificed and heart, liver, spleen, lung, kidney, and tumor tissues from the representative mice in each group were sectioned into slices for H&E TUNEL staining analysis. The stained slices were examined using an optical microscope for detailed observations of histological changes in the organs.

### Measurement of liver and kidney function assay

For liver and kidney function analysis, adult male BALB/c mice were used. Five days following FeCO_3_ NPs (10 mg Fe ion/kg of mice) injection, blood was collected from the retro-orbital sinus at specified time points. Liver and kidney activity was assessed by measuring the serum ALT, ASP, BUN, CRE levels by using a Hitachi Type 717 automatic analyzer (Hitachi).

### Statistical analysis

All data are presented as the mean ± standard deviation, comparing groups using the student’s t test or one-way ANOVA using GraphPad Prism 5 Software (GraphPad Software, Inc., San Diego, CA, USA). A P value of < 0.05 was considered statistically significant. *P < 0.05; **P < 0.01; ***P < 0. 001. n = 3 number of mice was used for each in vivo treatment groups.

## Results and discussion

### Synthesis and characterization of FeCO_3_ NPs

**Fig. 1:**
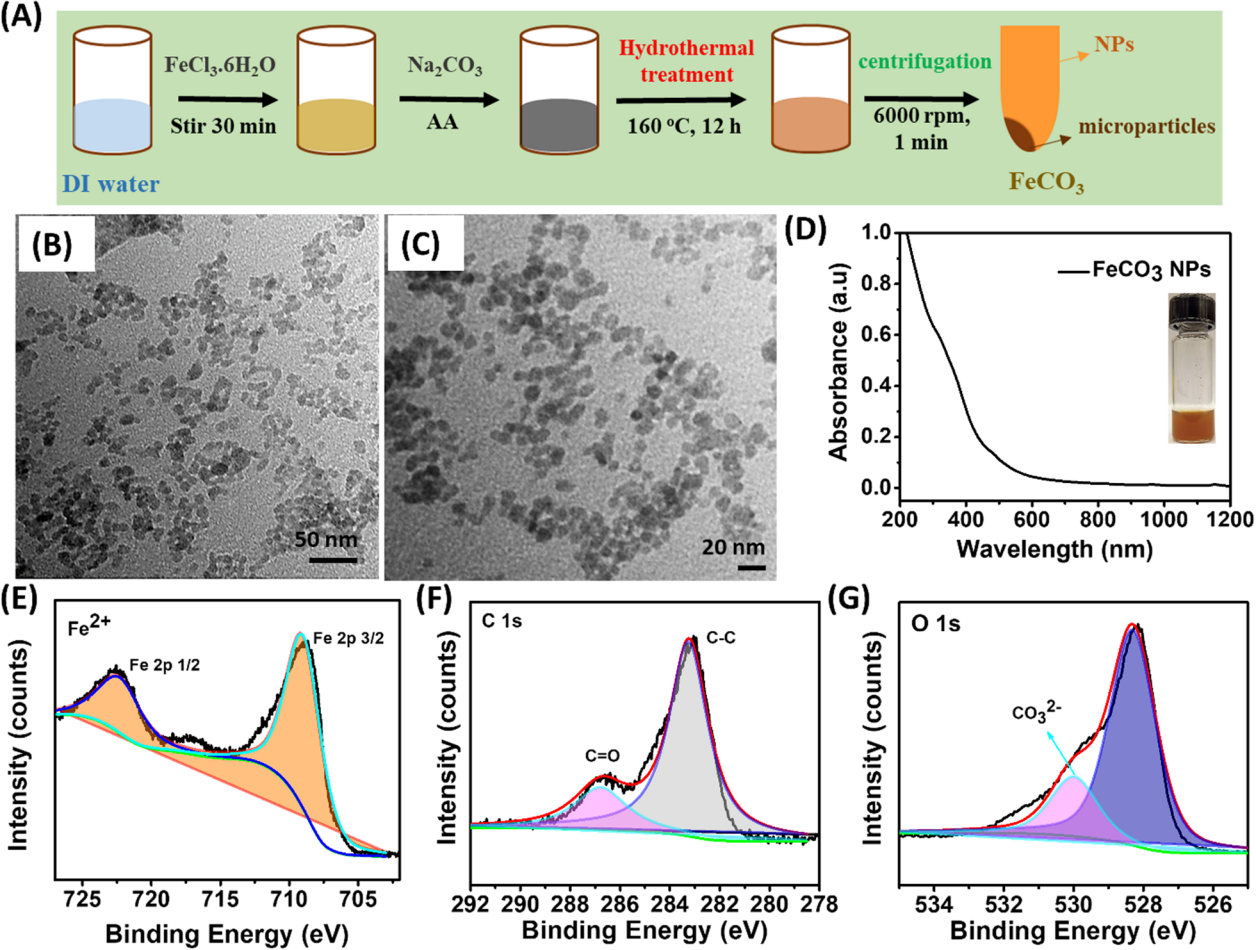
Synthesis and characterization of FeCO_3_ NPs. **A** schematic representation of FeCO_3_ NPs synthesis. **B**, **C** are respectively low and high magnification TEM images of FeCO_3_ NPs. **D** UV–visible-NIR absorption spectra of FeCO_3_ NPs, aqueous dispersion of FeCO_3_ NPs shown in the inset. **E**–**G** HR-XPS spectra of Fe, C and O, respectively

**Fig. 2:**
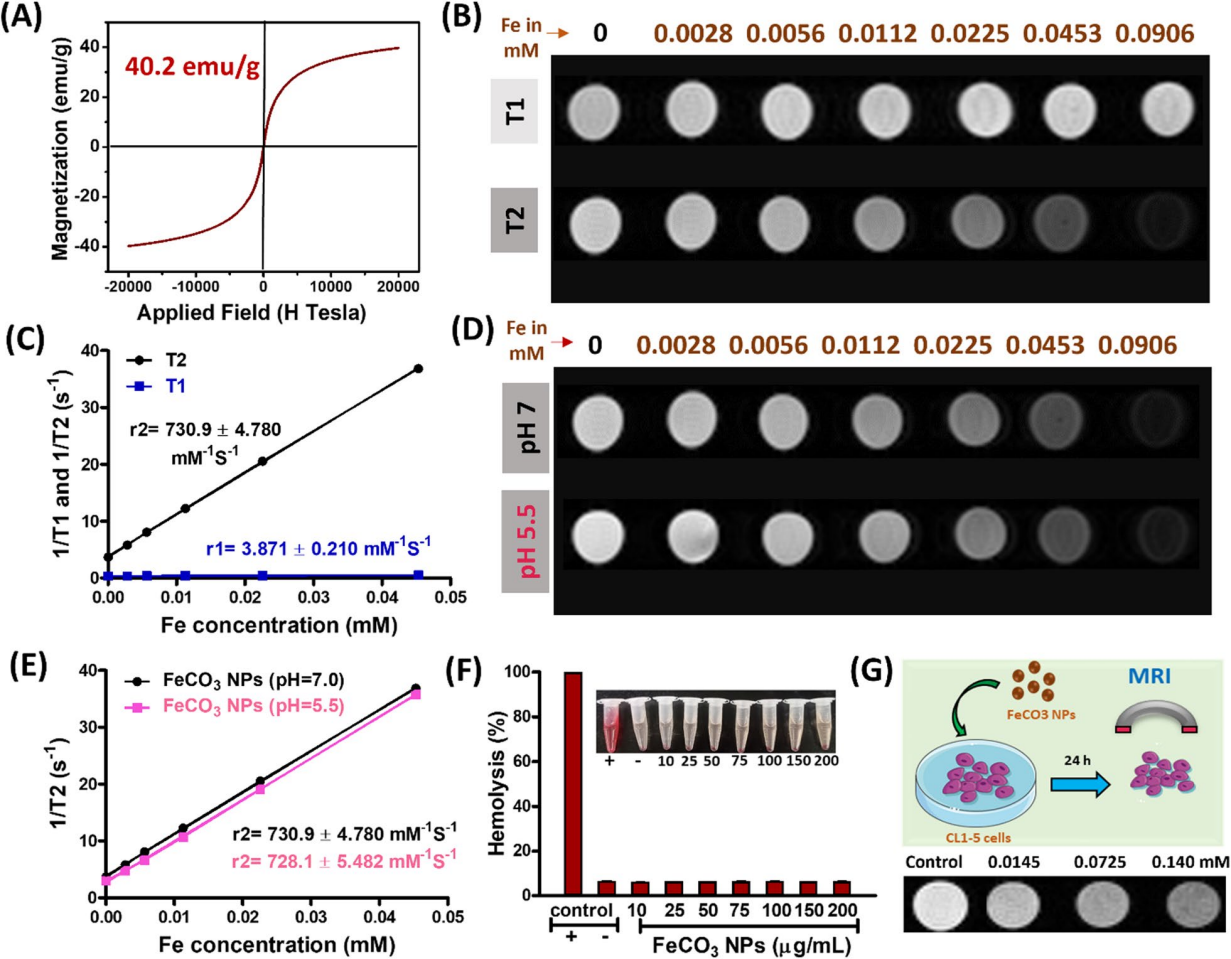
Structural and functional properties of FeCO_3_ NPs. **A** Magnetic hysteresis loops of FeCO_3_ NPs at 300 K. **B** T1 and T2-weighted magnetic contrast properties of FeCO_3_ NPs in aqueous media. **C** r1 and r2 values of FeCO_3_ NPs with respect to the Fe ion concentration. **D** T2 weighted MRI phantoms of FeCO_3_ NPs at pH 5.5 and 7.0. **E** r2 relaxivity values of FeCO_3_ NPs at pH 5.5 and 7.0. **F** Hemolysis assay of FeCO_3_ NPs at different concentrations, H_2_O and 0.9% NaCl were respectively used as negative and positive controls (inset show optical images of assay aliquots). **G** T2-weighted MR contrast ability of lung cancer cells in vitro, pre and post-24 h incubation of FeCO_3_ NPs

Ultra-fine FeCO_3_ NPs with an average size of ~ 10 nm were synthesized using a previously reported procedure with a slight modification [30]. The FeCO_3_ NPs synthesis scheme is shown in detail in Fig. 1A. Previous studies used FeCO_3_ at the micrometer scale. To achieve smaller FeCO_3_ NPs, we applied a simple centrifugation separation technique (6000 rpm, 5 min) following hydrothermal treatment, resulting in FeCO_3_ NPs in a supernatant and FeCO_3_ microparticles in residue. Microparticle size in the residue was estimated to be > 500 nm, as shown in Additional file 1: Fig. S1. After centrifuging, the resulting FeCO_3_ NPs were analyzed using high resolution transmission electron microscopy (HR-TEM) to assess morphology, distribution, and average size. As shown in Fig. 1B, C, the HR-TEM image of the FeCO_3_ NPs reveals that particles are uniform in size and spherical in shape. Notably, individual particle sizes of FeCO_3_ NPs from TEM analysis are ~ 7 to 8 nm. UV–visible-NIR absorption spectra of the FeCO_3_ NPs exhibits good optical absorption in the spectral region and the inset shows the optical image of the FeCO_3_ NPs dispersed in an aqueous solution (Fig. 2D). X-ray photoelectron spectroscopy (XPS) analysis confirmed the structural and phase composition of the resulting FeCO_3_ NPs. An XPS survey spectrum range of 1200–0 eV shows the clear presence of iron (Fe), carbon (C) and oxygen (O) in the FeCO_3_ NPs (Additional file 1:Fig. S3). High resolution (HR)-XPS spectra of Fe shows two binding energies at 709 and 722 eV, respectively corresponding to states Fe 2p 3/2 and Fe 2p 1/2, representing Fe in + 2 oxidation state in FeCO_3_ NPs (Fig. 1E). HR-XPS spectra of C1s shows two individual peaks with binding energies of 283.1 and 286.7 eV (Fig. 1F). The peak of the lower binding energy is assigned to correspond to C–C or C–H bonding, while the peak of the higher binding energy is assigned to correspond to the carbonate (${\text{CO}}_{3}^{2 - }$) peak. However, the peak of the higher binding energy is somewhat shifted to the lower binding energy, possibly due to the interaction of carbonate species with lower oxidation state bonding [31]. As shown in Fig. 1G, the O 1 s spectra region exhibited two distinct peaks with binding energies of 528.2 and 530 eV, respectively corresponding to the –OH and carbonate [32]. Overall, HR-XPS analysis confirmed the final composition of the synthesized nanoparticles are in FeCO_3_ form.

### Magnetic properties of FeCO_3_ NPs

For MRI applications, contrast agents should possess superparamagnetic behavior and induced relaxation. Thus, the magnetization of FeCO_3_ NPs was measured at 40.2 emu per gram of Fe (Fig. 2A). The reversible hysteresis curves indicate the superparamagnetic behavior of FeCO_3_ NPs at room temperature, allowing for biomedical imaging applications. FeCO_3_ NPs also shows good response to an external magnet (Additional file 1: Fig. S4). Subsequently, the T1 and T2-weighted MR contrast ability of the FeCO_3_ NPs aqueous solutions was examined using varying concentrations of FeCO_3_ NPs (with respect to Fe) under a 9.4 T animal MR scanner. As shown in Fig. 2B, T2 weighted MR phantom images of FeCO_3_ NPs showed significant signal attenuation upon increasing the Fe concentration from 0.0028 mM to 0.0906 mM (Fe concentration in FeCO3 NPs was estimated by using an inductively coupled plasma-mass-spectrometry technique). In contrast, no obvious changes were observed in the T1-weighted MR phantom images even at higher concentrations under otherwise identical conditions. The data clearly indicates that FeCO_3_ NPs exhibited T2-weighted MR contrast properties, causing the signal intensity of T2-weighted images to gradually decrease as Fe concentrations increase. Plots of signal intensity versus inversion time for the T1/T2-relaxation times with respect to the Fe concentrations are shown in Fig. 2C. The corresponding relaxation rates (r1 = 1/T1 and r2 = 1/T2) of the FeCO_3_ NPs exhibit a linear relation to the Fe concentration. The calculated r1 and r2 values for FeCO_3_ NPs are respectively 3.871 and 730.9 mM^−1^ S^−1^. Metal carbonates such as MnCO_3_ nanocrystals exhibit greater contrast in acidic pH than in neutral pH by decomposing the MnCO_3_ to Mn^2+^ ions. To determine whether this occurs in the FeCO_3_ NPs, we investigated the pH-dependent MR contrast properties of FeCO_3_ NPs both in neutral (pH = 7.0) and acidic media (pH = 5.5). As shown in Fig. 2D, no significant changes were observed in the T2-weighted phantom images, indicating no apparent dissolution of FeCO_3_ NPs. To further we examined the structural and optical changes of FeCO_3_ NPs by treating with acetate buffer (pH = 5.6). No significant changes were observed in the optical spectra of FeCO_3_ NPs before and after incubation in acidic condition (acetate buffer, pH = 5.6) for a period of 60 min. Besides, no changes were observed in solution color which reveals that no possible decomposition of FeCO_3_ NPs at pH = 5.6 (Additional file 1: Fig. S5A). Subsequently, structural stability of FeCO_3_ NPs via XPS analysis, shown in (Additional file 1: Fig. S5B–D). Even after treating with acetate buffer (pH = 5.6), high resolution XPS analysis shown a Fe^2+^ and C peaks corresponding to the FeCO_3_ composition which clearly indicates that Fe^2+^ of FeCO_3_ NPs is not oxidized into Fe^3+^ at pH 5.6. One of the plausible reasons for the stability of FeCO_3_ NPs is as follows, FeCO_3_ decomposition via decarbonated sorbent pathway leads to the formation of Fe_3_O_4_, Fe/C and CO_2_ (Eq. 1) which can re-absorb CO_2_ to form FeCO_3_ again (Eq. (2)) [33, 34]. The quick decarbonation and carbonation reactions in FeCO_3_ facilitates good stability of FeCO_3_ without decomposition. However, exact mechanism remains unclear, which is certainly a good topic for future investigations.

1 $$ {\text{6FeCO}}_{{3}} \left( {\text{s}} \right) \, \to {\text{ 2Fe}}_{{3}} {\text{O}}_{{4}} \left( {\text{s}} \right) \, + {\text{ Fe}}/{\text{C}}\left( {\text{s}} \right) \, + {\text{ 5CO}}_{{2}} \left( {\text{g}} \right). $$

2 $$ {\text{2Fe}}_{{3}} {\text{O}}_{{4}} \left( {\text{s}} \right) \, + {\text{ Fe}}/{\text{C}}\left( {\text{s}} \right) \, + {\text{ 5CO}}_{{2}} \left( {\text{g}} \right) \, \to {\text{ 6FeCO}}_{{3}} \left( {\text{s}} \right). $$

As a result, we did not notice any changes in the r2 values in either medium, and the r2 values of FeCO_3_ NPs are 730.9 ± 4.8 mM^−1^ S^−1^ in pH = 7.0 and 728.1 ± 5.5 mM^−1^ S^−1^ in pH = 5.5 (Fig. 2E). Notably, we compared the T2-weighted contrast abilities of FeCO_3_ NPs with FDA-approved clinical contrast agents such as Resovist^®^ (r_2_ = 374.6 ± 12.6 mM^−1^ s^−1^) [35] and Friedx (r_2_ = 307 mM^−1^ s^−1^) [36]. The r2 relaxation value of FeCO_3_ NPs is 1.95, 2.3 times higher than that of Resovist^®^, Friedx and FeREX standards under 9.4 T MR system. As discussed earlier, surface coating ligands on FDA-approved contrast agents such as carboxydextran (on Resovist^®^) [37] and dextran polymer (on Friedx) [37] might result in lower r2 values than that of the FeCO_3_ NPs. In T2 weighted imaging, T2 contrast agents interact with surrounding water molecules by inducing a local magnetic field. This displays as a relatively “dark” area on T2 weighted images [38]. Most of the carbonate-based nanostructures are porous in nature so which believed to be enabled more water molecules absorb into the pores. Thus, the abundance of more water molecules on FeCO_3_ NPs surface may help to promote fast proton exchange with water molecules results an enhanced r2 values. The colloidal stability properties of the FeCO_3_ NPs were studied in different media including water, phosphate buffered saline (PBS) and Dulbecco's Modified Eagle's Medium (DMEM). No significant aggregations were observed in the different media, indicating excellent colloidal stability (Additional file 1: Fig. S6). Prolonged incubation of FeCO_3_ NPs in aqueous solution also had no effect on stability, with no apparent changes in the absorption spectra (Additional file 1: Fig. S7).

### In vitro biocompatibility studies and uptake of FeCO_3_ NPs

Prior to in vitro and in vivo applications, we evaluated the cytotoxicity and hemocompatibility of the FeCO_3_ NPs. The in vitro cytotoxicity test was performed on normal cells (human fetal lung fibroblast cells (MRC-5, 1 × 10^4^) using a standard Cell Counting Kit-8 (CCK-8) assay at various concentrations of FeCO_3_ NPs (0, 10, 25, 50, 75, 100, 150, 200 μg/mL). Results show that FeCO_3_ NPs had no significant toxic effect on cells during prolonged incubations (24 h and 48 h), and cell viability remained 100% even at higher concentrations (200 μg/mL) (Additional file 1: Fig. S8). This strongly indicates the FeCO_3_ NPs possess excellent biocompatibility in vitro with no toxicity. No hemolysis activity was detected for different concentrations of FeCO_3_ NPs, even at 200 μg/mL, indicating that high compatibility with blood cells (Fig. 2F, inset shows assay aliquots images). Water and PBS were respectively used negative and positive controls. Compared to the negative control, a slight degree of hemolysis was found on the FeCO_3_ NPs, but the hemolysis percentage remained below 3% at experimental NPs concentrations, verifying their excellent hemocompatibility [39]. Targeting cancer cells with NPs could thus effectively improve the cells’ background contrast for MR imaging. Several previous studies have shown that NPs can accumulate in tumors via passive targeting (the so-called Enhanced Permeability and Retention (EPR) effect) based on the difference between specific pathophysiological characteristics of tumors vs healthy tissues [40, 41]. To confirm the cancer cell uptake and passive targeting ability of FeCO_3_ NPs to further validating MRI performance, CL1-5-luc/GFP lung cancer cells were treated with different concentrations of FeCO_3_ NPs (0, 0.0145, 0.0725, 0.140 mM, respective to Fe ions) and the in vitro MRI performance was evaluated using a 9.4 T animal MRI system scanner (Fig. 2G). After 24 h of incubation, cells treated with FeCO_3_ NPs darkened significantly with the increased concentration of NPs corresponding to the T2-weighted contrast with respect to the control group, indicating FeCO_3_ NPs were successfully internalized in the cancer cells via passive targeting/EPR effect.

### T2-weighted MR imaging of in vivo lung tumors

**Fig. 3:**
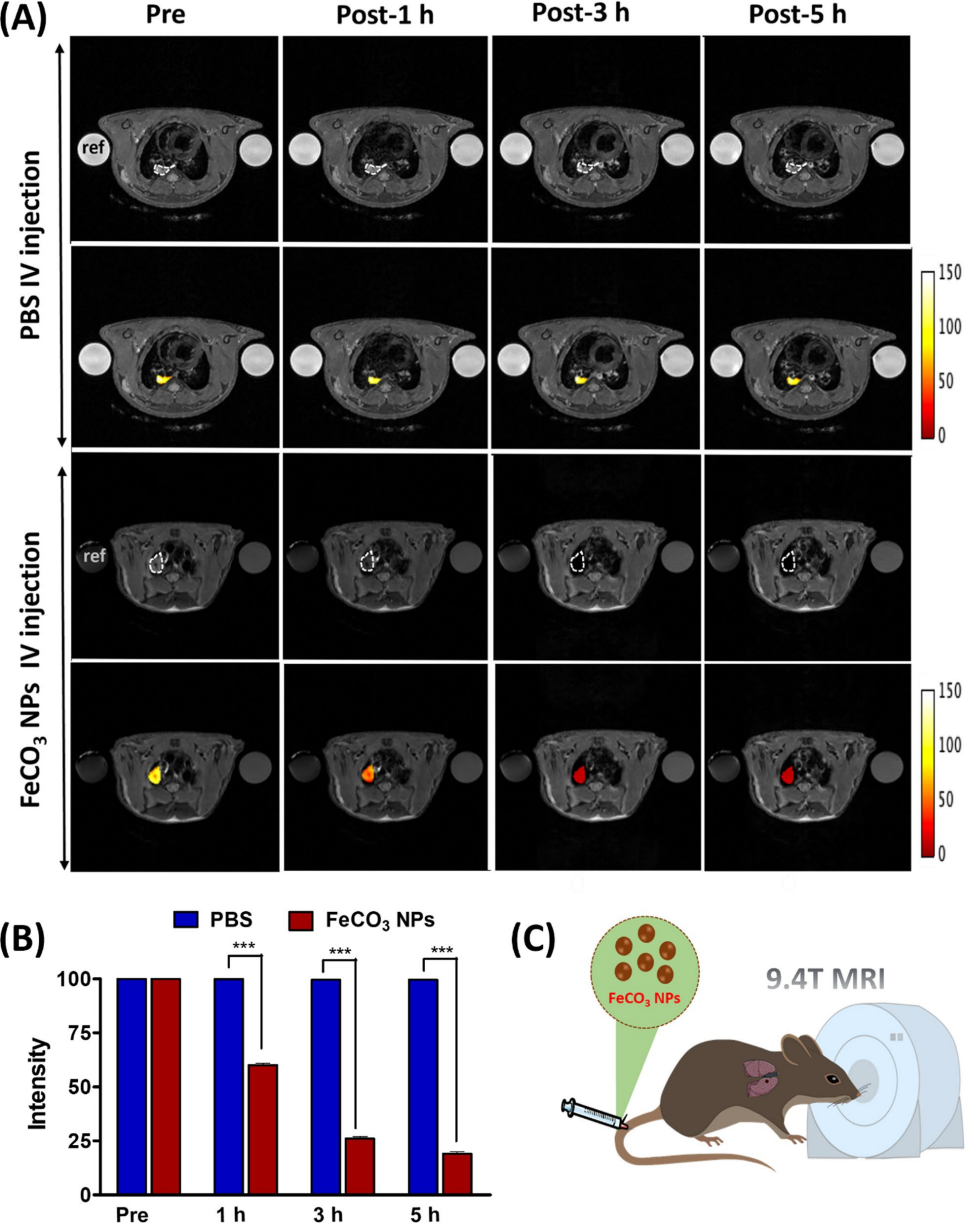
In vivo MR contrast properties of FeCO_3_ NPs. **A** in vivo (n = 3) T2-weighted MR images of lung tumors pre and post-1 h, 3 h, 5 h injection of PBS and FeCO_3_ NPs (dose of NPs; 10 mg Fe ion/kg, i.v injection, MRI scans were performed on a 9.4 T MRI scanner, circle/pseudo color indicates the tumor region). **B** Kinetic plot of in vivo contrast ability of PBS and FeCO_3_ NPs, data is represented as mean ± SD; ***P ≤ 0. 001. **C** Schematic representation of in vivo MR imaging of lung tumors using FeCO_3_ NPs on a 9.4 T MRI scanner

**Fig. 4:**
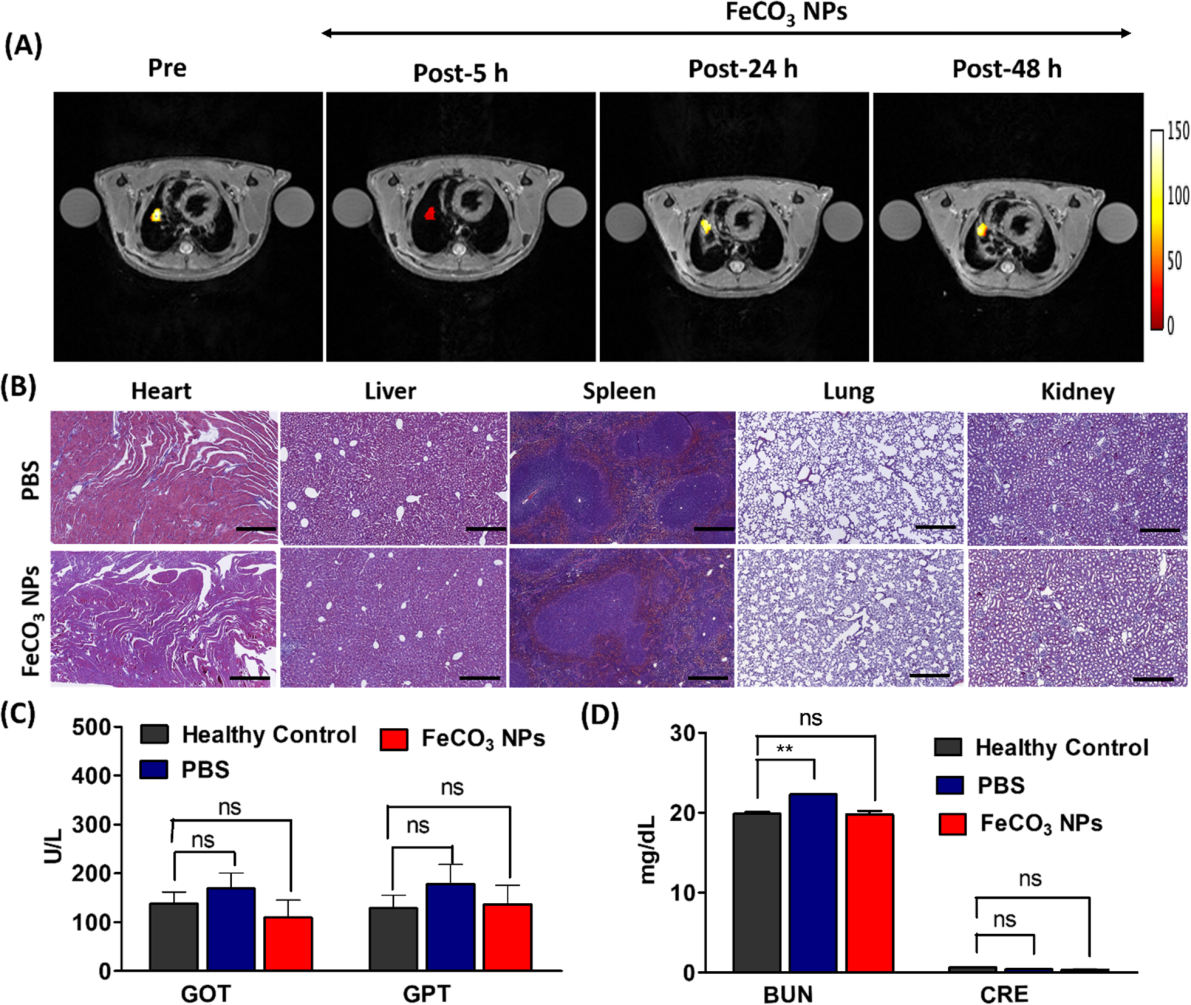
In vivo biocompatibility studies of FeCO_3_ NPs. **A** T2-weighted in vivo MR imaging of lung tumors 5 h, 24 h and 48 h post-injection (10 mg/kg of FeCO_3_ NPs per mice were administered intravenously, MRI scans were performed on a 9.4 T MRI scanner). **B** Histopathological observation of major organs including heart, liver, spleen, kidney, and lung of mice 5 days post-injection of FeCO_3_ NPs (10 mg/kg of FeCO_3_ NPs per mice were administered intravenously, scale bars are 500 μm). **C**, **D** respectively show liver and kidney function analysis of in vivo mice 5-days post injection of PBS and FeCO_3_ NPs (10 mg of Fe ion/kg of mice). n = 3 mice were used for all experiments. ns: ≥ 0.05; *P ≤ 0.05; **P ≤ 0.01; ***P ≤ 0.001

As a proof of concept, we investigated the T2-weighted MR imaging ability of FeCO_3_ NPs on in vivo lung tumor mice models. Lung tumors were implanted via direct injection of luciferase expressed CL1-5-luc/GFP lung cancer cells (10 µL, 5 × 10^4^) into the lung (experimental setup shown in Additional file 1: Fig. S9A). Two weeks after tumor implantation, lung tumors were identified using IVIS imaging technique (Additional file 1: Fig. S10B). After successful lung tumor formation, FeCO_3_ NPs were administered intravenously (at a dose of 10 mg/kg of mice) for real-time tracking of in vivo lung tumors using a 9.4 T MR imaging system and compared against a PBS control group. Tumor cells can uptake the NPs via the enhanced permeation and retention (EPR) effect through which NPs are trapped in the tumor tissue through a leaky tumor vasculature and are then retained in the tumor bed due to reduced lymphatic drainage, resulting in successful therapeutics and imaging applications [42–44]. Due to the EPR effect, we observed enhanced T2-weighted MR contrast of in vivo lung tumors after intravenous administration of FeCO_3_ NPs. Briefly, we acquired T2-weighted MR images of in vivo mice at different time points for FeCO_3_ NP (pre-injection, post 1 h, 3 h, 5 h) and PBS (pre-injection, post 1 h, 3 h, 5 h). As shown in Fig. 3A, FeCO_3_ NPs began to accumulate in the tumor at 1 h following injection, resulting in a gradual darkening effect corresponding to the T2-weighted contrast observed in the tumor region. Moreover, this T2-weighted contrast in the lung tumor region increased with post FeCO_3_ NPs injection time from 1 to 5 h. No significant changes in tumor contrast were observed in the PBS group, and all the post injection PBS groups (1 h, 3 h, 5 h) showed the same degree of contrast. To provide statistically significant results, n = 3 mice were used for the in vivo MR imaging investigation of both the FeCO_3_ NPs and PBS groups (Additional file 1: Figs. S10, S11). We also include color map images in Fig. 3A for clear visibility of tumor contrast in the lung region. We further quantified the signal changes in the lung tumor region through a close analysis of the regions of interest (ROIs) and compared differences between the pre- and post- FeCO_3_ NPs injection, as well as with the PBS control group (Fig. 3B). Specifically, 55% of the darkening was observed at 1 h and more than ≥ 85% of darkening signal was observed at 3 h and 5 h post-injection of FeCO_3_ NPs with respect to the pre-injection group. Moreover, the T2 contrast effect of FeCO_3_ NPs is well maintained in the lung tumor region (1–5 h post-injection), much longer than that of the Gd based complex small molecules (half-life of about several minutes in small animals) [39, 45]. The longer in vivo retention time of the T2 effect on FeCO_3_ NPs allows for the effective location and study of tumors to obtain and evaluate important pathological information. In contrast, no significant changes were noticed in the lung tumor region of the PBS control group. The schematic representation of the in vivo MR imaging of lung tumors on FeCO_3_ NPs is shown in Fig. 4C. Overall, in vitro*,* and in vivo results strongly suggests that the passive targeting of FeCO_3_ NPs could promote the internalization of more NPs into the tumor cells and exhibit excellent T2-weighted MR contrast for successful visualization/location of in vivo lung tumors.

### In vivo biocompatibility studies of FeCO_3_ NPs

Most of the NP-based drugs limit their use in practical/clinical applications due to concerns about chronic accumulation and patient safety [46]. Thus, the USFDA strictly requires NPs to be biodegradable and metabolized or excreted from the body after their intended biomedical applications [46, 47]. Thus, we examined the in vivo potential toxicity and excretion of FeCO_3_ NPs after intravenous administration into the mice models. As shown in Fig. 4A, MR imaging data reveals the remarkable tumor darkening effect corresponds to the T2 contrast observed after 5 h following FeCO_3_ NPs injection. As post-injection time increased to 24 h and 48 h, the tumor contrast gradually increased which strongly indicating that NPs were slowly excreted from the tumor site in the days following injection. We also observed the same phenomenon in the liver region, where accumulation of FeCO_3_ NPs corresponds to the T2-weighted darkening effect at the initial administration and liver contrast later increased which also strongly confirms the excretion of NPs after a prolonged time following administration (Additional file 1: Fig. S12). Later, hematoxylin and Eosin (H&E) staining analysis results show no histological differences in the major organs 5-days following FeCO_3_ NPs injection with respect to the control PBS group (Fig. 4B). H&E and in vivo MR imaging studies, indicating good renal clearance from the body following potential MR imaging applications. Serum biochemistry assays were also conducted to investigate the intrinsic toxicities of FeCO_3_ NPs on major organs such as liver and kidney functions (Fig. 4C, D). We analyzed liver function parameters/indicators such as aspartate aminotransferase (AST/GOT), alanine aminotransferase (ALT/GPT) and kidney function indicators such as serum creatinine (CRE) and blood urea nitrogen (BUN). Five days after FeCO_3_ NPs injection, no significant changes in liver and kidney functions were observed and all functional values were within the range of the healthy control and PBS control groups, with data respectively shown in Fig. 4C, D. In addition, we also performed the Perl's Prussian blue analysis to detect the presence of iron in tissue samples (Additional file 1: Fig. S13). After 5-day post i.v. injection of FeCO_3_ NPs, no traces of Fe were observed in the major organs indicating that the successful excretion of NPs after the treatment. Importantly, particle size of NPs are greatly influence on in vivo blood clearance profiles and clearance from body. It was reported that smaller size (5 and 20 nm) of NPs showed rapid clearance from the blood than the larger size (50 nm) NPs after post i.v. injection [48, 49]. Therefore, it was expected that the present smaller size of FeCO_3_ NPs can be able to clear rapidly from blood since the particle size of FeCO_3_ NPs are < 20 nm as a result no noticeable side effects or toxicities to major organs. Overall, our biocompatibility results clearly indicate that the as-synthesized FeCO_3_ NPs exhibited good cyto/hemo compatibility with no obvious toxicity in mice, making them potential candidates for future biomedical MR imaging applications for early diagnosis of lung cancer.

## Conclusion

In summary, we have successfully developed the first biocompatible, ultra-small FeCO_3_ NPs for T2-weighted MR imaging of in vivo lung tumors. FeCO_3_ NPs exhibited a uniform size distribution, good dispersibility and excellent stability in both aqueous and biological media. Moreover, aqueous solutions of FeCO_3_ NPs displayed remarkable T2-weighted MR contrast with a r2 value of 730.9 ± 4.8 mM^−1^ S^−1^, which is respectively 1.95 and 2.3 times higher than the clinically approved contrast agents Resovist^®^ and Friedx at an identical conditions (under same 9.4 T MR system). Taking advantage of the remarkable T2 contrast abilities, we further demonstrate remarkable T2 contrast ability (≥ 85%) in the tumor region after 5 h post intravenous administration of FeCO_3_ NPs. Moreover, the in vivo T2 contrast ability of the FeCO_3_ NPs is well maintained in the lung tumor region (1–5 h, after post injection of NPs) compared to Gd-based complex small molecules (half-life of several minutes in small animals). Furthermore, in vitro and in vivo biosafety studies reveal the FeCO_3_ NPs are highly biocompatible and no significant toxic effects to the major organs. Overall, the proposed biocompatible FeCO_3_ NPs present as a potential candidate for real-time tracking of in vivo lung tumors in future clinical applications for early lung cancer diagnosis.

## Supplementary Information

**Additional file 1**: **Fig. S1**. TEM images of FeCO_3_ microparticles in the residue; **Fig. S2**. DLS size distribution spectra of FeCO_3_ NPs. **Fig. S3**. XPS survey spectrum of FeCO_3_ NPs; Figure S4. FeCO_3_ NPs in the presence of an external magnetic field; **Fig. S5**. Colloidal stability of FeCO3 NPs in different meda; **Fig. S6**: Absorption spectra of FeCO_3_ NP aqueous solution before and after 30 days; **Fig. S7**: In vitro cell viability of FeCO_3_ NPs in MRC-5 normal cells; Figure S8: Lung tumor implantation setup; **Figs. S9 and S10**: In vivo T2-weighted MR images of mice models (n = 3) after injection of PBS and FeCO_3_ NPs respectively; **Fig. S11**: In vivo T2-weighted contrast of liver after injection of FeCO_3_ NPs; **Table S1**. Comparison of image modalities used in molecular imaging.

## Data Availability

All supporting data for this study are included in this published article and its additional information files. All the experimental protocols involving live animals were reviewed and approved by the Institutional Animal Care and Use Committee (IACUC No. 2021031802) of Chang Gung Memorial Hospital and were performed in accordance with the Animal Protection Regulations of the Council of Agriculture, Executive Yuan (R.O.C.) and the guidelines of the National Research Council (U.S.A.) for the care and use of laboratory animals.
