# Supplementary material for: Magnetic, biocompatible FeCO3 nanoparticles for T2-weighted magnetic resonance imaging of in vivo lung tumors
Source: J Nanobiotechnology. 2022 Mar 25;20:157. doi: 10.1186/s12951-022-01355-3 (PMC8952886; doi:10.1186/s12951-022-01355-3)
Supplement: Supplementary file 1 — Additional file 1: Fig. S1. TEM images of FeCO3 microparticles in the residue; Fig. S2. DLS size distribution spectra of FeCO3 NPs. Fig. S3. XPS survey spectrum of FeCO3 NPs; Figure S4. FeCO3 NPs in the presence of an external magnetic field; Fig. S5. Colloidal stability of FeCO3 NPs in different meda; Fig. S6: Absorption spectra of FeCO3 NP aqueous solution before and after 30 days; Fig. S7: In vitro cell viability of FeCO3 NPs in MRC-5 normal cells; Figure S8: Lung tumor implantation setup; Figs. S9 and S10: In vivo T2-weighted MR images of mice models (n = 3) after injection of PBS and FeCO3 NPs respectively; Fig. S11: In vivo T2-weighted contrast of liver after injection of FeCO3 NPs; Table S1. Comparison of image modalities used in molecular imaging. [file 12951_2022_1355_MOESM1_ESM.docx]

**Magnetic, Biocompatible FeCO_3_ Nanoparticles for T2-weighted Magnetic Resonance Imaging of in vivo Lung Tumors**

*Suresh Thangudu^†^, Chun-Chieh Yu^†^, Chin-Lai Lee^†^, Min-Chiao Liao^†^, Chia-Hao Su^†, ‡*^*

†Institute for Translational Research in Biomedicine, Kaohsiung Chang Gung Memorial Hospital, Kaohsiung 833, Taiwan.

‡Department of Biomedical Imaging and Radiological Sciences, National Yang Ming Chiao Tung University, Taipei 112, Taiwan.

*Corresponding author email: [chiralsu@gmail.com](mailto:chiralsu@gmail.com).

**Supplementary Figures**


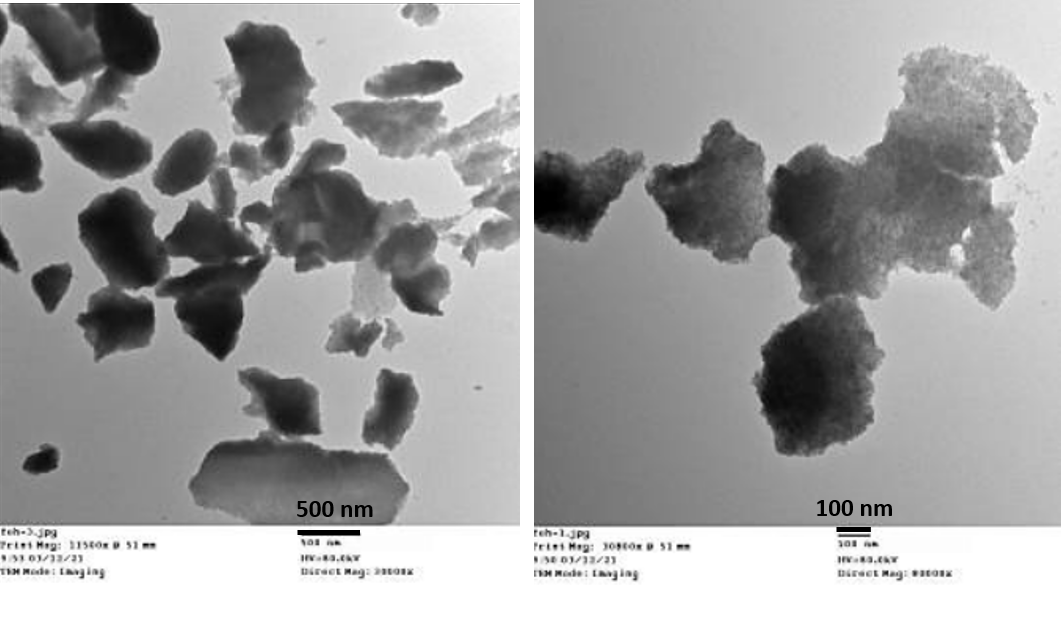


**Figure S1**. TEM images of FeCO_3_ microparticles in the residue.


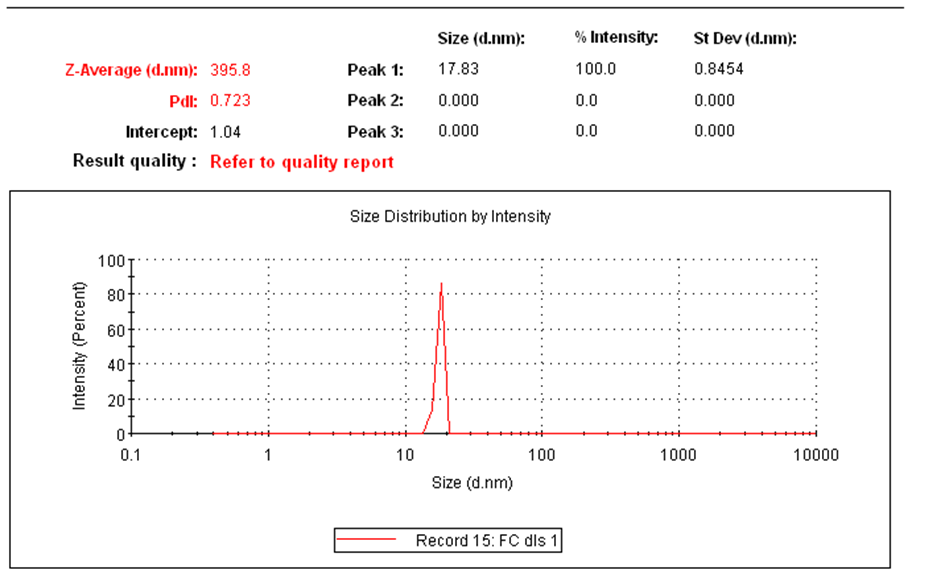


**Figure S2**. DLS size distribution spectra of FeCO_3_ NPs.

**Figure S3**. XPS survey spectrum of FeCO_3_ NPs.

**
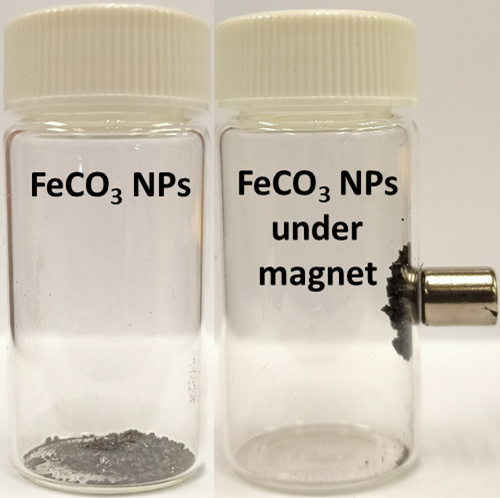
**

**Figure S4**. FeCO_3_ NPs in presences of external magnetic field.


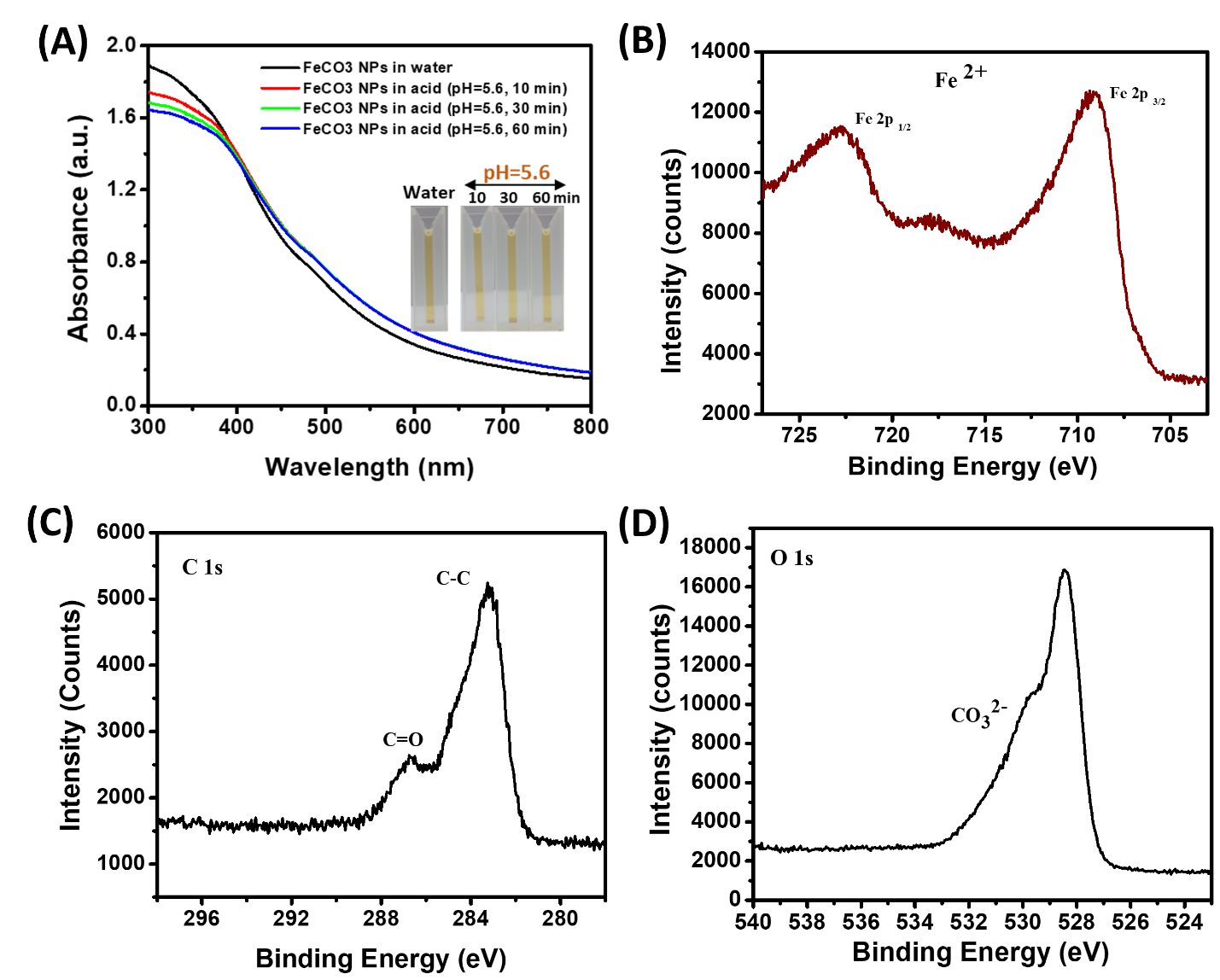


**Figure S5**. (A) Optical absorption spectra of FeCO_3_ NPs in acid (condition: pH 5.6, incubation time 10, 30, 60 min), inset shows the images of FeCO_3_ NPs in acid solution. (B), (C) and (D) HR-XPS of FeCO_3_ NPs after treating with acid (pH 5.6).


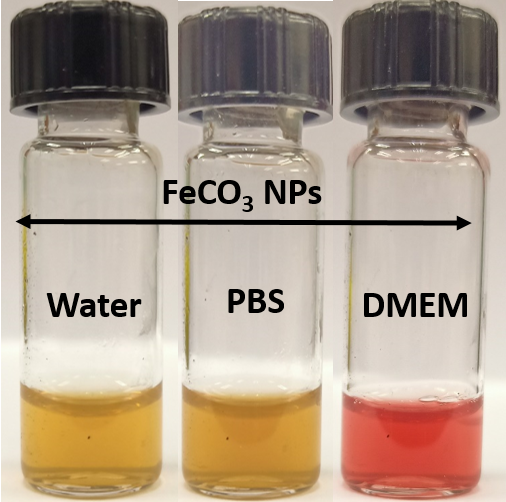


**Figure S6**. Colloidal stability of FeCO_3_ NPs in different medium (deionized water, H_2_O; Phosphate-buffered saline, PBS; Dulbecco's Modified Eagle Medium, DMEM). FeCO_3_ NPs were dispersed in different medium and obtained the optical images.

**Figure S7**. Absorption spectra of FeCO_3_ NPs aqueous solution before and after 30 days.

**Figure S8**. *In vitro* cell viabilities of FeCO_3_ NPs in MRC-5 normal cells (24h and 48h incubation of NPs with different concentration). ns: P ≥0.05; *: P ≤0.05; **: P ≤0.01; ***: P ≤ 0.001.


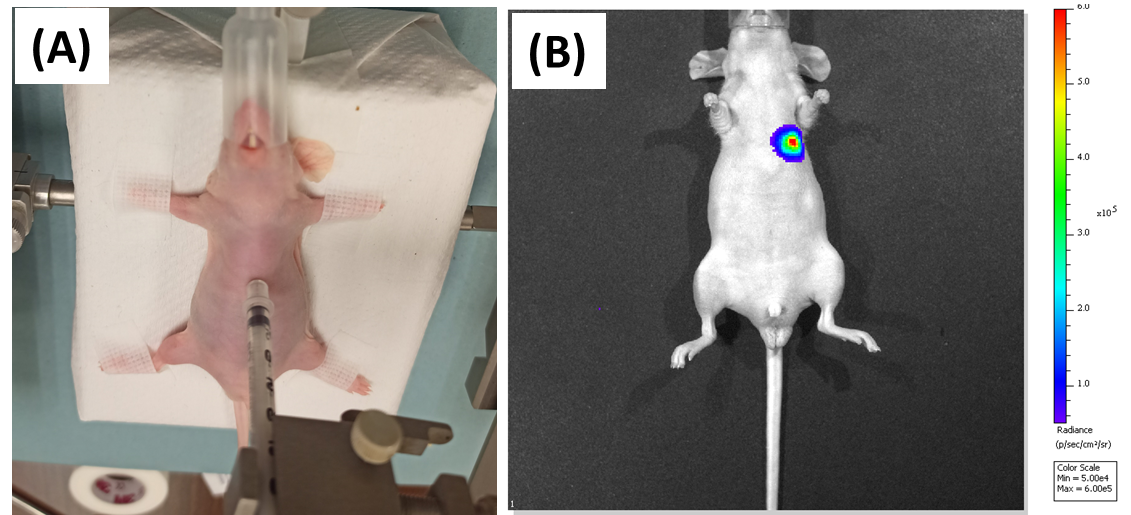


**Figure S9**. Lung tumor implantation setup (A) experimental setup for implantation of in vivo lung tumors via direct injection of cancer cells into lungs. (B) Identification of successful formation of lung tumor via IVIS imaging technique (emission at 560 nm).


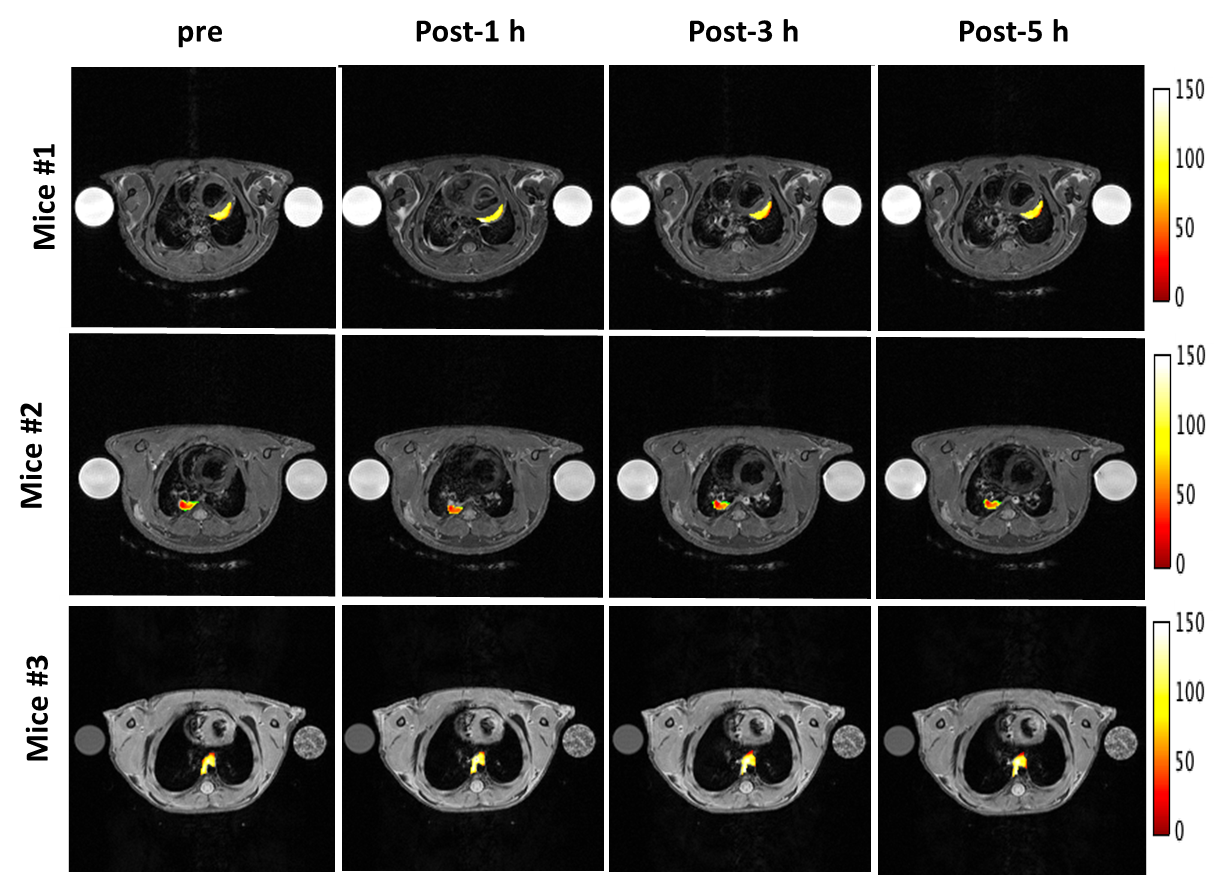


**Figure S10**. *In vivo* T2 weighted MR images of mice models (n=3) after injection of PBS. Experimental condition; PBS (100 μl, 1X PBS) were intravenously administered to the lung tumor mice model. After pre and post injection time points (1h,3h, 5h), mice were examined under 9.4T animal MR scanner. Pseudo color represents the tumor region.


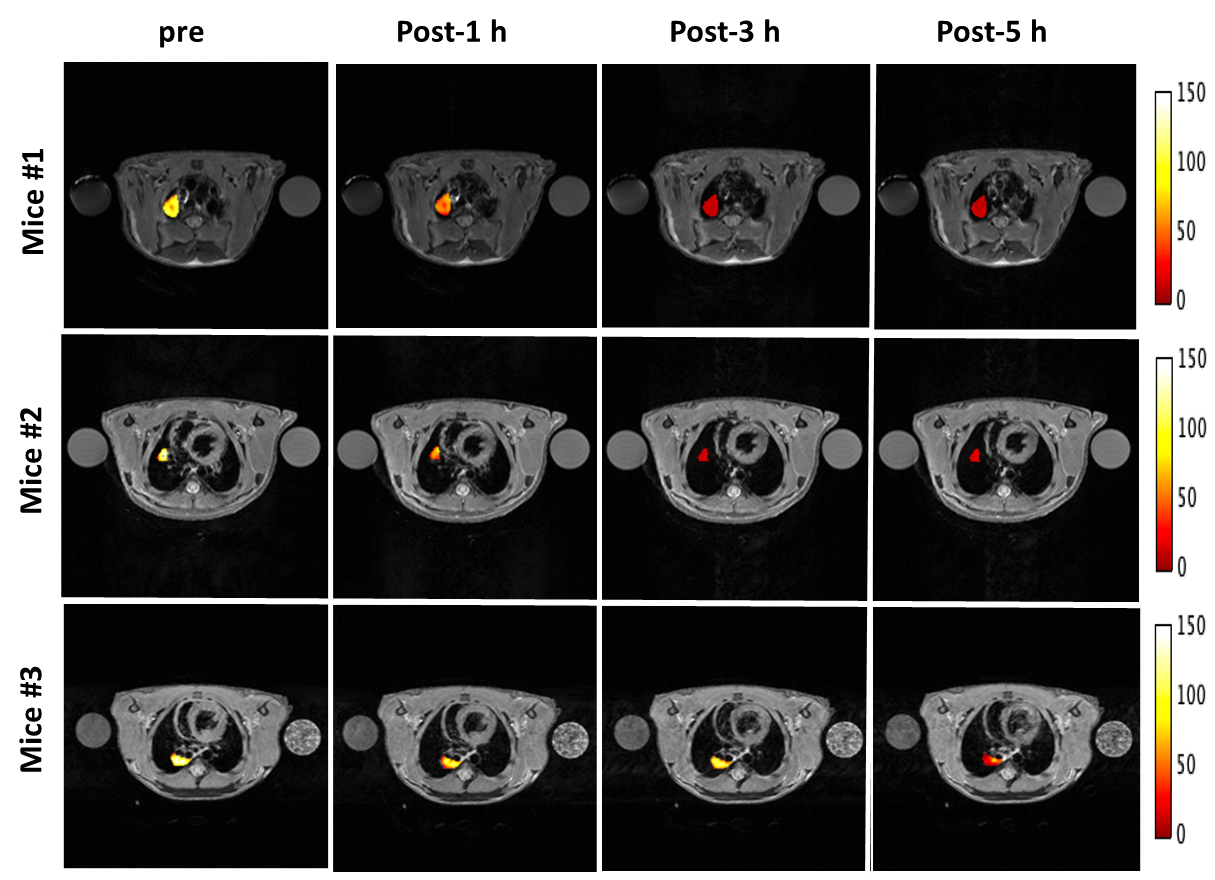


**Figure S11***. In vivo* T2 weighted MR images of mice models (n=3) after injection of FeCO_3_ NPs (10mg/kg of mice weight). Experimental condition; FeCO_3_ NPs (10mg of Fe ion/kg of mice weight) were intravenously administered to the lung tumor mice model. After pre and post injection time points (1h,3h, 5h), mice were examined under 9.4T animal MR scanner. Pseudo color represents the tumor region.


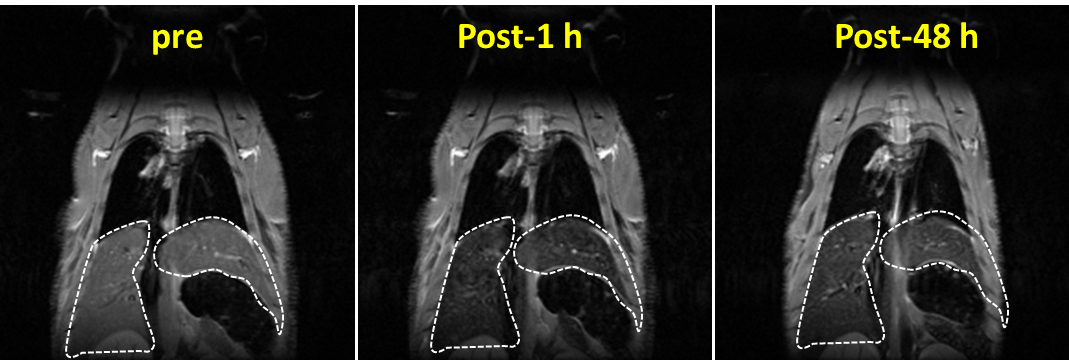


**Figure S12**. *In vivo* T2 weighted contrast of liver after injection of FeCO_3_ NPs. Experimental condition; FeCO3 NPs (10mg of Fe ion/kg of mice weight) were intravenously administered to the lung tumor mice model. After pre and post injection time points (1h, 48h), mice were examined under 9.4T animal MR scanner (white circled line represents the liver).


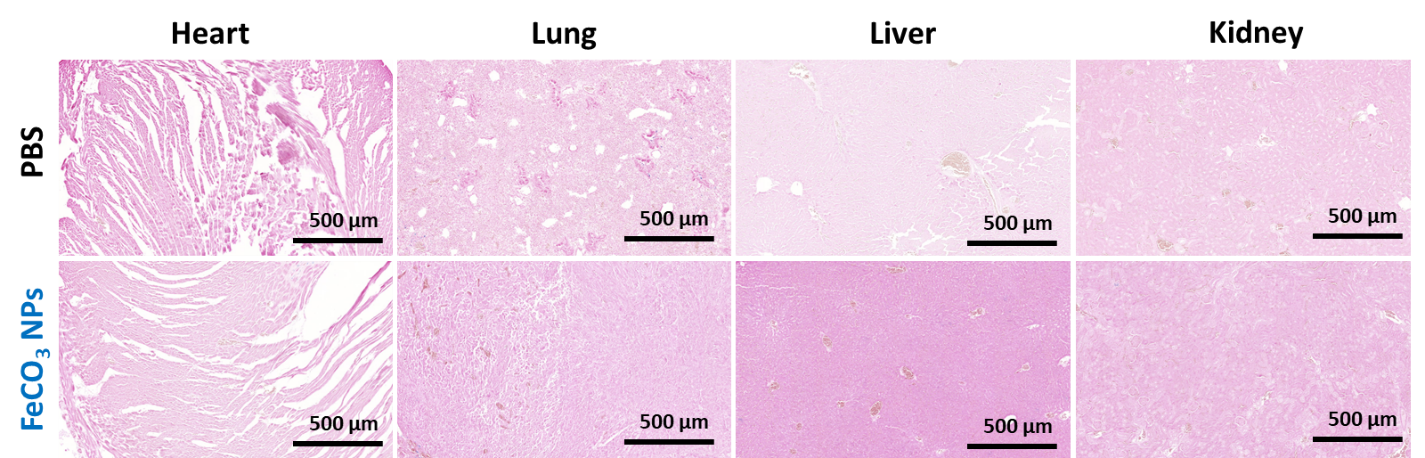


**Figure S 13**. Perl's Prussian blue stain for iron detection in major organs (experimental condition; 10 mg Fe/kg, post 5 day of i.v. injection).

**Table S1.** Comparison of image modalities used in molecular imaging.

| **Imaging**  **system** | **Spatial resolution** | **Depth** | **Scan**  **time** | **Cost(USD)** | **Clinical use** |
| --- | --- | --- | --- | --- | --- |
| **Nuclear**  **medicine** | 1~2 mm | No limit | Minutes ~ hours | PET: >300,000  SPECT: 100,000~300,000 | Yes |
| **Computed tomography** | 50μm | No limit | Minutes | 100,000~300,000 | Yes |
| **MRI** | 10~100μm | No limit | Minutes ~ hours | >300,000 | Yes |
| **Optical**  **system** | 1μm | < 400μm | Seconds ~ minutes | >300,000 | In develpoment |
| **Ultrasound** | 50μm | Milli  -meters | Minutes | 100,000~300,000 | Yes |
